# Impact of Sintering Aid Type and Content on the Mechanical Properties of Digital Light Processing 3D-Printed Si_3_N_4_ Ceramics

**DOI:** 10.3390/ma17235830

**Published:** 2024-11-27

**Authors:** Qing Qin, Lin Han, Gang Xiong, Zihan Guo, Junwei Huang, Yujuan Zhang, Zhen Shen, Changchun Ge

**Affiliations:** 1 Institute of Powder Metallurgy and Advanced Ceramics, School of Materials Science and Engineering, University of Science and Technology Beijing, Beijing 100083, China; qinqing20@mails.ucas.ac.cn (Q.Q.);; 2 State Key Laboratory of Multimodal Artificial Intelligence Systems, Beijing Engineering Research Center of Intelligent Systems and Technology, Institute of Automation, Chinese Academy of Sciences, Beijing 100190, China

**Keywords:** DLP 3D printing, sintering aids, pressure sintering, mechanical property

## Abstract

Digital light processing (DLP) 3D-printed Si₃N₄ ceramics, known for their exceptional performance, offer distinct advantages in meeting the high-strength and complex structural demands of industries such as aerospace, semiconductors, healthcare, automotive, energy, and machinery. However, due to Si₃N₄’s strong chemical stability, low diffusion rate, low self-sintering ability, and high melting point, achieving densification under conventional sintering conditions is challenging. As a result, sintering additives are essential to promote the sintering process, lower the sintering temperature, improve densification, and enhance performance. In this study, 45 vol% Si₃N₄ slurries were prepared using DLP 3D printing technology, incorporating nine different combinations of sintering additives, including aluminum oxide (Al_2_O_3_), yttrium oxide (Y_2_O_3_), and aluminum nitride (AlN), in various ratios with Si_3_N_4_. The slurries were then sintered at 1800 °C for 2 h under a 1 MPa N_2_ atmosphere. Additionally, the phase composition, microstructure, grain distribution, and crack propagation of the materials. The results showed that a Si_3_N_4_ to Al_2_O_3_ and Y_2_O_3_ ratio of 95:2.5:2.5 produced elongated β-Si_3_N_4_ grain structures and enhanced density, achieving a maximum Vickers hardness of 12.88 ± 0.52 GPa. Additionally, the synergistic toughening effect of the rod-like β-Si_3_N_4_ grains and sintering aids significantly improved the fracture toughness of the Si_3_N_4_ ceramic matrix, with a flexural strength of 540.63 ± 10.05 MPa and a fracture toughness of 4.92 ± 0.07 MPa·m^1/2^. This study lays the foundation for the future application of 3D-printed Si_3_N_4_ ceramics, optimization of sintering aid combinations at different ratios, and performance enhancement in extreme environments.

## 1. Introduction

**Figure 1:**
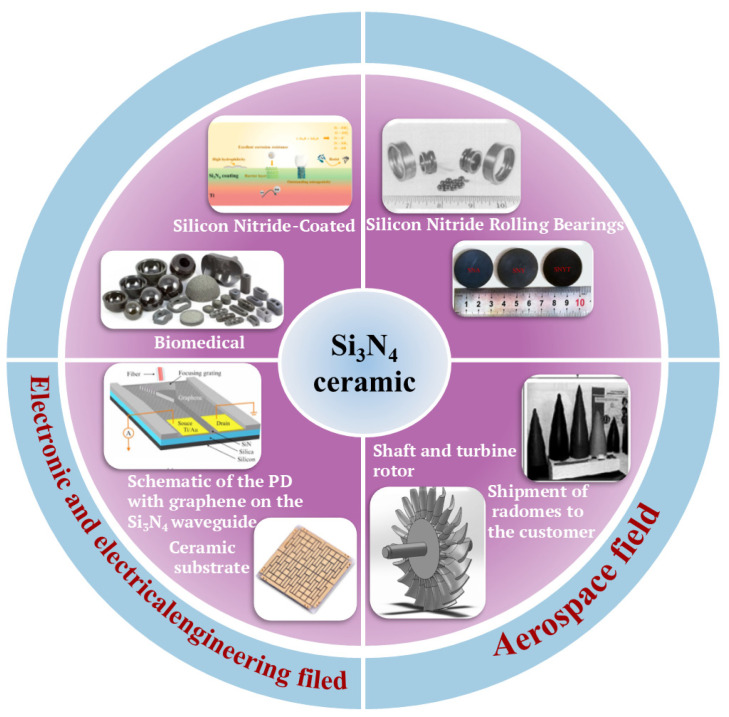
Different application fields of Si_3_N_4_ ceramics.

Si_3_N_4_ is an essential structural ceramic celebrated for its exceptional properties, including wear and corrosion resistance, high flexural strength, fracture toughness, creep resistance, and hardness [1]. Si_3_N_4_ components are widely used in various fields, including mechanical engineering, electronics, electrical engineering, medicine, and aerospace. Notable applications include cutting tools [2], rolling bearings [3], ceramic substrates [4], integrated circuits [5], bone integration scaffolds [6], dental implant coating materials [7], missile radomes [8], turbine rotors, and turbine blades [9], as shown in Figure 1. Many applications of Si_3_N_4_ necessitate complex geometric structures; however, traditional ceramic processing methods, such as hot pressing and mold extrusion, are often constrained to simple axisymmetric shapes.

DLP 3D printing technology represents a mature Additive Manufacturing (AM) technique that provides a more efficient approach to producing ceramic components. DLP utilizes a ceramic slurry composed of ceramic powder, photosensitive resin, and initiators as raw materials for manufacturing ceramic products. Upon exposure to ultraviolet light of a specific wavelength for a few seconds, the ceramic particles bond together layer by layer, forming complex ceramic structures [10].

Recent studies have demonstrated that complex-shaped ceramics have been successfully fabricated using DLP 3D-printing technology, including oxide ceramics such as Al_2_O_3_ [11] and ZrO_2_ [12], as well as non-oxide ceramics like Si_3_N_4_ [13]. DLP technology integrates and projects entire print layers, achieving faster speeds, higher precision, and shorter production cycles. Components can be directly produced from Computer-Aided Design (CAD) files without the need for complex tooling, allowing for the creation of intricate shapes. This method is suitable for complex three-dimensional structures and requires minimal post-processing [14]. However, the produced preforms often exhibit high porosity, which typically necessitates elimination through sintering or pyrolysis to achieve densification [15].

Si_3_N_4_ has strong covalent bonds and a low self-diffusion coefficient for silicon and nitrogen, making it difficult to densify at high temperatures. As a result, sintering additives are essential during the process to promote sintering densification and regulate the microstructure of Si_3_N_4_ ceramics [16,17]. In the liquid-phase sintering process, the formation of β-Si_3_N_4_ grains involves particle rearrangement, solution-reprecipitation, and Ostwald ripening [18]. The composition, concentration, and viscosity of the liquid phase play a key role in modulating the microstructure and ceramic properties, such as enhancing fracture toughness and strength [19,20,21]. When sintering α-Si_3_N_4_ free powders, commonly used combinations include oxide sintering aids; these additives can be used individually or in combination [22]. Meng Li et al. fabricated Si_3_N_4_ ceramics using DLP technology and successfully obtained a slurry with the lowest viscosity (0.042 Pa·s at a shear rate of 30 s^−1^) and a higher single-layer curing depth (47.9 ± 1.2 µm) by coating Al_2_O_3_-Y_2_O_3_ sintering additives onto the Si_3_N_4_ powder. The relative density and bending strength of the processed Si_3_N_4_ ceramics reached their maximum values of 85.4% ± 0.3% and 149.2 ± 1.9 MPa, respectively [23]. Ya-Ru Wu et al. successfully fabricated porous Si_3_N_4_ ceramics by combining DLP technology with the pore-forming agent method. Using Al_2_O_3_-Y_2_O_3_ as a sintering additive, they produced porous ceramics with a 40 vol% volume fraction, achieving a bending strength of 435.87 MPa [24]. Rufei Chen et al. used digital light processing (DLP) 3D printing technology and Al_2_O_3_-Y_2_O_3_ as a sintering additive to successfully fabricate high-performance broadband microwave-transparent Si_3_N_4_-SiO_2_ composite ceramics. At a sintering temperature of 1350°C, the bending strength of the Si_3_N_4_-SiO_2_ ceramics reached 77 ± 5 MPa [25]. Chong Tian fabricated porous Si_3_N_4_ ceramics using digital light processing (DLP) technology and successfully produced high-performance broadband microwave-transparent Si_3_N_4_ ceramics with Al_2_O_3_-Y_2_O_3_ as a sintering additive. The resulting slurry exhibited low viscosity (2.38 Pa·s at a shear rate of 30 s^−1^) and high curing depth (51.2 ± 4.6 μm), with the final ceramics achieving a bending strength of 329.7 MPa [26]. The advantage of the Al_2_O_3_-Y_2_O_3_ system is its potential to form crystalline compounds, such as yttrium aluminum garnet (YAG, Y_3_A_l5_O_12_), but the final strength was relatively low. This may be related to factors such as the ratio and type of sintering additives or the sintering temperature. Further investigation also focused on commonly used non-oxide sintering additives [22]. Zhen-Kun Huang and Anatoly Rosenflanz achieved fully dense Si_3_N_4_ ceramics with high strength at elevated temperatures by using intermediate liquid compositions in the (Y, La)_2_O_3_-AlN system as additives sintering. These ceramics exhibit strength of 1150 MPa at 1200 °C and maintain a room temperature toughness of 7 MPa·m^1/2^ [27]. Ya-Ru Wu and Jun-Hong He used Y_2_O_3_ and AlN as sintering aids and excellent physical and mechanical properties have been achieved in Si_3_N_4_–SiC (w) composites [28,29]. They found that with the increase in AlN addition, the total porosity of porous Si_3_N_4_ ceramics tends to decrease, thereby increasing the flexural strength [30]. Many methods exist for sintering ceramics. Gas pressure sintering, which imposes no restrictions on shape or size, is particularly suited for ceramic samples produced by DLP 3D printing technology. The N_2_ atmosphere positively influences the densification and grain growth of sintered Si_3_N_4_ ceramics [31]. Overall, these studies indicate that by precisely controlling the type and proportion of sintering additives, as well as the sintering process parameters, the performance of DLP-printed Si_3_N_4_ ceramics can be significantly improved, particularly in terms of bending strength, density, and optimization of microstructure.

This study investigates the effects of three commonly used non-oxide and oxide sintering aids on the mechanical properties of Si_3_N_4_ produced by DLP 3D printing. Given the significant impact of sintering aid content on the resulting liquid phase, this paper examines nine different additive formulations, a topic that has received relatively limited research attention. It also compares their effects on the performance, microstructure, and toughness enhancement mechanisms of Si_3_N_4_ ceramics sintered under 1 MPa pressure in an N_2_ atmosphere.

## 2. Experiment

### 2.1. Raw Materials

Using an average particle size D50 of 1.03 μm, the main phase is α-Powder of Si_3_N_4_ (α-Phase > 93%, oxygen content < 0.8%) from China’s Jinan ZhongCostanbo New Materials Co., Ltd. The sintering aids (Y_2_O_3_, 5.01 g/cm^3^, Aladdin, China; Al_2_O_3_, 4 g/cm^3^, Aladdin, China; AlN, 3.26 g/cm^3^, Aladdin, China). Hexamethylene diacrylate (HDDA, 1.01 g/cm^3^, Macklin, China) and Trimethylolpropane triacrylate (TMPTA, 1.1 g/cm^3^, Macklin, China) are used as photosensitive resin monomer. Phenylbis (2,4,6-trimethylbenzoyl) phosphine oxide (BAPO, 1.17 g/cm^3^, Aladdin, China) is a photoinitiator. BYK-9076 (Beijing Ten Dimensions Technology Co., Ltd., Beijing, China) is used as a dispersant.

### 2.2. Fabrication

The sample formulas with different sintering aids and different contents of the same sintering aid are shown in Table 1.

Firstly, untreated Si_3_N_4_ powder (including sintering aids) and ethanol were mixed in a 1:2 ratio and milled at 300 r/min for 2 h. The resulting slurries were dried in an oven for 8 h, then grounded for 30 min, and then powders were obtained by passing through the 80 mesh. Meanwhile, TMPTA and HDDA were mixed in a beaker to obtain a photosensitive resin premix. The premix and sieved Si_3_N_4_ powder were milled together at 300 r/min for 2 h with a 2:1 ball-to-powder mass ratio. Then, Dispersants and photoinitiators were added, and the mixture was milled again at 300 r/min for 2 h to prepare the slurry, which was then stored in a dark brown bottle.

**Figure 2:**
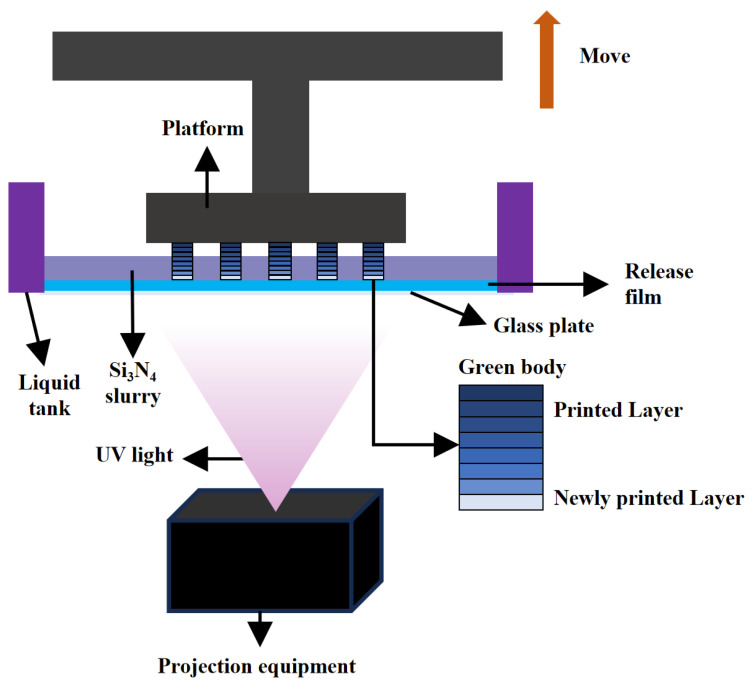
DLP 3D printing process.

The green body was produced using a bottom-up DLP printer (AutoCera, Beijing Ten Dimensions Technology Co., Ltd., China). Initially, the computer converted the 3D model into 2D images along the Z-axis and sent them to the printer. Then, the printer is prepared and set to use a 405 nm ultraviolet wavelength to cure photosensitive resins or ceramic slurries. Next, the Si_3_N_4_ slurry is poured into the liquid tank, and a blade evenly spreads the slurry onto the print platform to ensure consistent layer thickness. Each layer of ceramic slurry is selectively cured with ultraviolet light for 6 s, resulting in a thickness of 10 μm per layer. After each layer is cured, the print platform is lowered, and fresh slurry is added to print the next layer, building the entire 3D model layer by layer. After printing is complete, the excess slurry is removed, and post-processing is performed to obtain the final Si_3_N_4_ object, as shown in Figure 2. After the excess slurry was removed, the final Si_3_N_4_ object was obtained.

The sintering and debonding processes of Si_3_N_4_ ceramics are detailed in the Supplementary Materials.

### 2.3. Characterization

Phase identification was carried out using an X-ray diffractometer (XRD, miniflex600, Japan) at a scanning speed of 10°/min with Cu Kα radiation (λ = 1.54Å). The microstructure of the sintered samples was characterized using a field emission scanning electron microscope (FE-SEM, Regulus 8100, Leo Corporation, Germany) at an accelerating voltage of 15 kV. The density of the samples was determined using the Archimedes method, and the relative density was calculated from the ratio of the theoretical density to the measured density (Secozhun Mayzum, MZ-AE1200-D43, Shenzhen, China). To evaluate the microstructure and mechanical properties, the standard test bars were printed with dimensions of 3 mm × 4 mm × 40 mm and scaled proportionally based on the shrinkage rate during the printing process. The flexural strength of each sample was measured from three test bars using a ceramic mechanical testing machine (CDW, Changchun Chaoyang Test Instrument Co., Ltd., Jilin, China) with a three-point bending method, a loading speed of 0.5 mm/min, and a span length of 10 mm. The hardness and fracture toughness of the samples were measured using the indentation method (MH-6, Everone Co., Ltd., Shanghai, China), with a load of 98 N and a dwell time of 15 s. The indentation and crack lengths were obtained by scanning, and the final Vickers hardness and fracture toughness were calculated from the average values of more than three notches, with fracture toughness (KIC) calculated using Formula (1):

(1) $$K_{\mathrm{IC}}=0.016{\left(\frac{E}{H}\right)}^{0.5}\left(\frac{P}{c^{1.5}}\right)$$

where E is the elastic modulus of the material, H is the projected hardness number, P is the Vickers load, and c is the radial crack length measured from the center of the indentation to each of the four crack tips [32].

## 3. Results and Discussion

### 3.1. Sintered Body, Phase Composition and Microstructure

**Figure 3:**
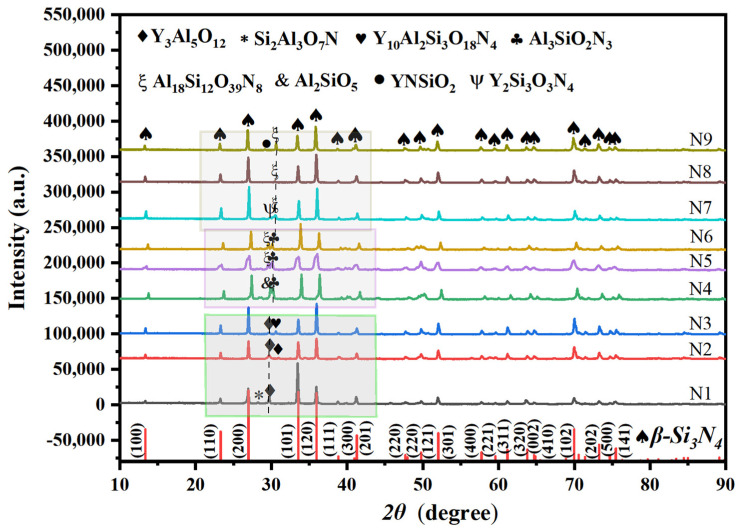
XRD patterns of obtained Si_3_N_4_ ceramic samples sintered at 1800 °C for 2 h with different samples.

Figure 3 presents the XRD patterns of Si_3_N_4_ ceramics after sintering. This study aims to investigate the effects of different contents and types of sintering additives on the phase transformation of Si_3_N_4_ during liquid-phase sintering. The XRD results indicate that samples N1 to N9 underwent a phase transformation from α-Si_3_N_4_ to β-Si_3_N_4_, with most diffraction peaks exhibiting high intensity and narrow full width at half maximum, suggesting a high degree of crystallinity in the ceramic samples. This reflects a reduction in disorder within the crystals, an increase in grain size, and a decrease in crystal defects such as dislocations and vacancies.

Samples N1, N2, and N3 contain not only the main phase Si_3_N_4_ but also trace amounts of Y_3_Al₅O₁_2_ (YAG), Si_2_Al_3_O₇N, and Y₁₀Al_2_Si_3_O_8_N_4_. At a low additive content of 5 wt%, the samples are primarily composed of YAG and Si_2_Al_3_O₇N phases. As the additive content increases to 10 wt%, the thermodynamic stability of the system changes. The Y_2_Si_3_O_3_N_4_ phase may become unstable in the presence of high concentrations of Y_2_O_3_ or Al_2_O_3_ and is likely to transform into thermodynamically more stable phases. The Si_2_Al_3_O_7_N phase disappears, while the amount of YAG phase correspondingly increases. When the additive content is further raised to 15 wt%, the Y_10_Al_2_Si_3_O_8_N_4_ phase appears. This occurs because, during high-temperature sintering, Y_2_O_3_ and Al_2_O_3_ react with a nitrogen source to form aluminum nitride (e.g., AlN), and they also react with Si_3_N_4_, ultimately forming aluminum–silicon nitride composite phases. During high-temperature sintering, some reactants melt to form different liquid phases, indicating interactions among the original Si_3_N_4_, Y_2_O_3_, Al_2_O_3_, and a small amount of SiO_2_ [33].

In samples N4, N5, and N6, the sintering additives are AlN and Al_2_O_3_. At low concentrations, Al_2_O_3_ reacts with Si_3_N_4_ during high-temperature sintering to form a small amount of the silicoaluminate phase AlSi_2_O₅, while Si_3_N_4_, AlN, and Al_2_O_3_ react to create Al_3_SiO_2_N_3_. AlN primarily supplies nitrogen and works alongside Al_2_O_3_ to facilitate densification. As the amount of sintering additives increases, the nitrogen atmosphere provides sufficient nitrogen sources, while excess AlN and Al_2_O_3_—especially at higher ratios—-supply adequate aluminum, oxygen, and nitrogen, promoting the formation of the nitrogen-rich phase Al_18_Si_12_O_39_N_8_. The reason may be insufficient reaction conditions, which can lead to incomplete solid solution reactions or suboptimal sintering temperature and time, or atmosphere, hindering the successful formation of the Sialon phase. Additionally, the lack of liquid phase formation among the sintering additives may result in precipitation, leading to an increase in porosity and defects, which, in turn, causes insufficient densification of the material.

In Figure 3, samples N7, N8, and N9 incorporate AlN and Y_2_O_3_ as sintering additives. The XRD patterns reveal that when a small amount of AlN and Y_2_O_3_ reacts with Si_3_N_4_, it forms a minor quantity of the Y_2_Si_3_O_3_N_4_ phase, primarily located at the grain boundaries of Si_3_N_4_, which enhances the material’s densification and high-temperature performance. Furthermore, Y_2_Si_3_O_3_N_4_ interacts with AlN to produce the byproduct phase Al_18_Si_12_O_39_N_8_. As the concentration of sintering additives increases, the Y_2_Si_3_O_3_N_4_ phase gradually diminishes. When the additive content reaches 15 wt%, excess AlN and Y_2_O_3_ react with the silicon source in a nitrogen atmosphere to form YNSiO_2_, resulting in decreased sintering performance [28].

**Figure 4:**
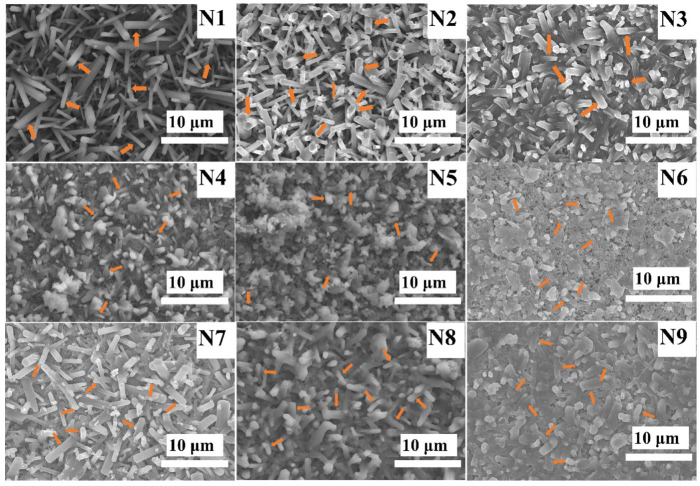
SEM micrographs of Si_3_N_4_ ceramics with different samples.

**Figure 5:**
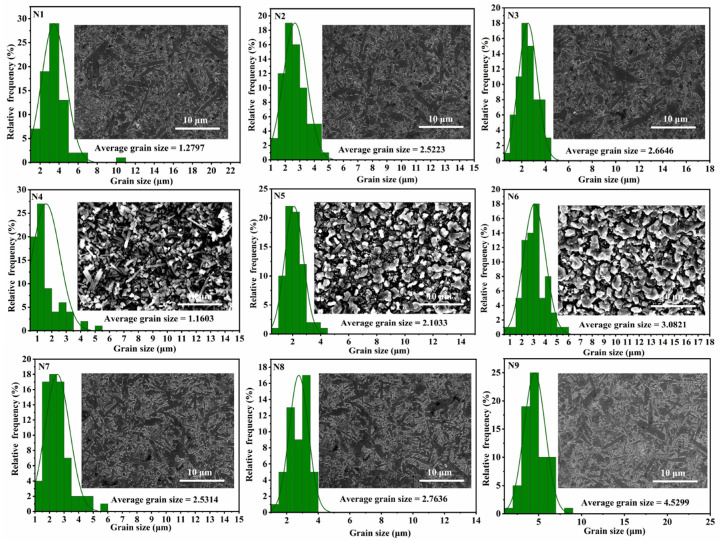
SEM micrographs and grains diameter distributions of Si_3_N_4_ ceramics after ion etching for 300 s for different samples.

To enhance the observation of grain morphology and arrangement in the Si_3_N_4_ samples, diamond abrasives of varying sizes were employed for grinding, followed by a meticulous polishing process. Figure 4 illustrates the SEM images of Si_3_N_4_ ceramics prepared at 1800 °C with different types and concentrations of sintering additives. Meanwhile, Figure 5 shows the corresponding SEM images, the relative frequency of the particle size distribution, and the fitted curve obtained after 300 s of ion etching. From the samples N1, N2, and N3 shown in Figure 4 (indicated by orange arrows), it is evident that the β-Si_3_N_4_ grains display elongated rod-like or fibrous structures, with lengths significantly exceeding their widths. This results in predominantly crossed morphologies in most areas. These intersecting grains effectively impede crack propagation during fracture, thereby enhancing the material’s fracture resistance and improving overall uniformity, which ensures outstanding mechanical properties in multiple directions [34]. The presence of relatively coarse rod-like structures in the figure may be attributed to the Ostwald ripening effect [35], in which smaller grains are preferentially consumed by larger grains through the liquid phase, increasing the overall grain size. According to Figure 5, approximately 40% of the grain size distribution for samples N1, N2, and N3 lies within the range of 2.25–4.0 μm. As the concentrations of Y_2_O_3_ and Al_2_O_3_ sintering additives increase, the particle size exhibits a noticeable upward trend. This suggests that an appropriate amount of sintering additives can improve material flow, promote the formation of a more uniform microstructure, and result in finer rod-like Si_3_N_4_ grains, as seen in sample N1. Excessively high levels of sintering additives may lead to undesirable grain growth, mainly because an excess of additives promotes the formation of a liquid phase, and the presence of the liquid phase typically accelerates grain growth. During the sintering process, the liquid phase provides more diffusion pathways between grains, facilitating grain enlargement. Additionally, a high concentration of additives may disrupt the microstructural stability of the material [36], as observed in samples N2 and N3.

Samples N4, N5, and N6 show that β-Si_3_N_4_ does not fully exhibit the typical elongated needle-like or rod-like structure. As indicated by the yellow arrows in the figure, only a few small-diameter rod-like Si_3_N_4_ particles were detected, suggesting that the growth of these rod-like grains is incomplete. This suggests that the liquid phase generated by the sintering additives is insufficient; despite the phase transformation, the particles have not developed into rod-like β-Si_3_N_4_ [2]. According to the statistical results of grain diameter presented in Figure 5, β-Si_3_N_4_ grains account for approximately 40% of the diameter range from 1.25 μm to 3.25 μm, and the grain size exhibits an increasing trend. This phenomenon may be due to the excessive sintering additive Al_2_O_3_ reacting with AlN and interacting with a limited amount of Si_3_N_4_ at high temperatures, resulting in the formation of a liquid phase that facilitates grain interactions and merging, ultimately leading to grain agglomeration. Compared to samples N1-N3, it is clear that the use of Y_2_O_3_ as a sintering additive enhances the formation of elongated β-Si_3_N_4_ grains.

From samples N7, N8, and N9 in Figure 4, it is evident that the AlN-Y_2_O_3_ sintering additives also display rod-like structures, although these are less pronounced than those formed by the Y_2_O_3_-Al_2_O_3_ additives. This suggests that, compared to AlN, Al_2_O_3_ enhances the hardness, wear resistance, and chemical stability of Si_3_N_4_, making it more suitable for high-temperature and high-stress environments. Additionally, the results in Figure 5 indicate that as the content of sintering additives increases, the grain size also increases, with the β-Si_3_N_4_ rod-like structures formed using 15 wt% AlN-Y_2_O_3_ exhibiting the most notable morphological characteristics.

**Figure 6:**
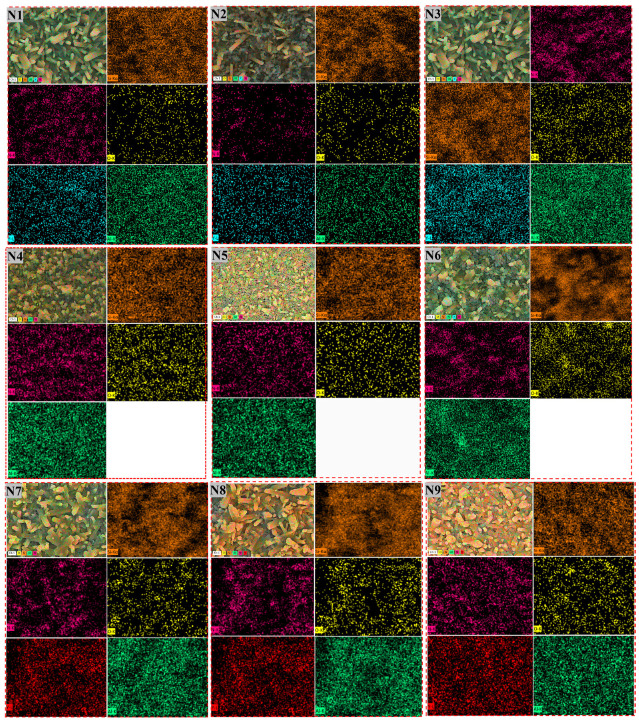
Mapping of Si_3_N_4_ ceramics with different samples.

To further investigate the elemental distribution in the microstructure of samples N1–N9, elemental mapping analysis was performed on each component sample, as shown in Figure 6. The analysis reveals that the distribution of N, O, Al/Al-Y, and Si elements in the Si_3_N_4_-based ceramic composites is relatively uniform, indicating that the added sintering additives effectively bonded with the Si_3_N_4_ matrix grains during the sintering process. As cooling progressed, an amorphous phase developed between the Si_3_N_4_ grains, which adversely affected the mechanical properties of Si_3_N_4_. Consequently, minimizing the content of the secondary phase along the grain boundaries is essential for improving the mechanical properties of Si_3_N_4_ [37].

### 3.2. Mechanical Properties of Si_3_N_4_ Ceramics

**Figure 7:**
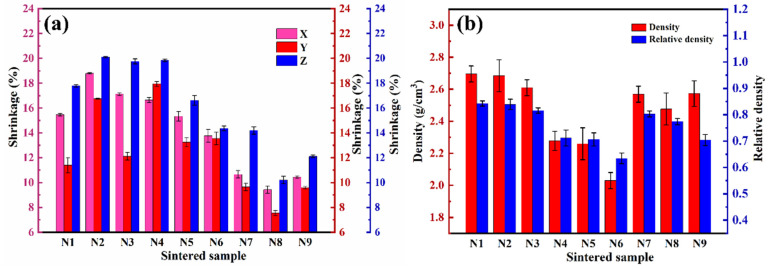
(**a**) Shrinkage rate and (**b**) relative density of different samples.

After debinding and sintering, the photosensitive resin in the green body decomposes, resulting in the formation of numerous pores. As the sintering process advances toward densification, the samples experience dimensional shrinkage in three-dimensional space. Figure 7a shows the shrinkage rate data for Si_3_N_4_ slurries prepared with various sintering additives after sintering at 1800 °C for 2 h. The results indicate that the amount of sintering additives has a minimal impact on the linear shrinkage of Si_3_N_4_ ceramics produced by DLP technology in all three directions, with the maximum shrinkage rate not exceeding 20%. Notably, the shrinkage rate in the Z-axis direction is significantly higher than that in the X/Y direction. This phenomenon is attributed to the layered structure characteristic of the DLP printing process, which creates larger gaps in the Z-axis direction compared to the X/Y directions. Furthermore, due to the influence of gravity, the shrinkage in the Z-axis direction is more pronounced, resulting in an overall shrinkage rate that surpasses that in the X/Y directions [23,38]. By comparing the shrinkage characteristics of the nine different sintering additives (N1 to N9), it is evident that the Y_2_O_3_-Al_2_O_3_ additives exhibit a slightly higher degree of shrinkage than the other groups. This suggests that the samples using additives N1 to N3 effectively eliminated pores during the sintering process, leading to a greater level of densification. As shown in Figure 7b, sample N1 achieved the highest density, reaching 84.25%.

**Figure 8:**
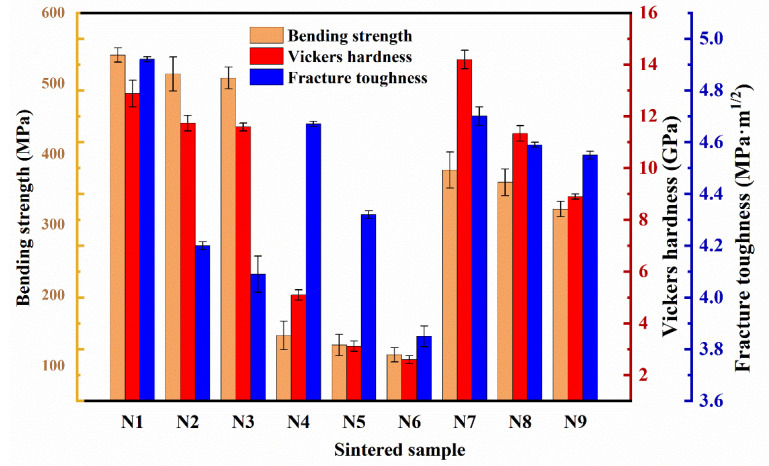
The mechanical performance of sintered samples with different samples.

**Figure 9:**
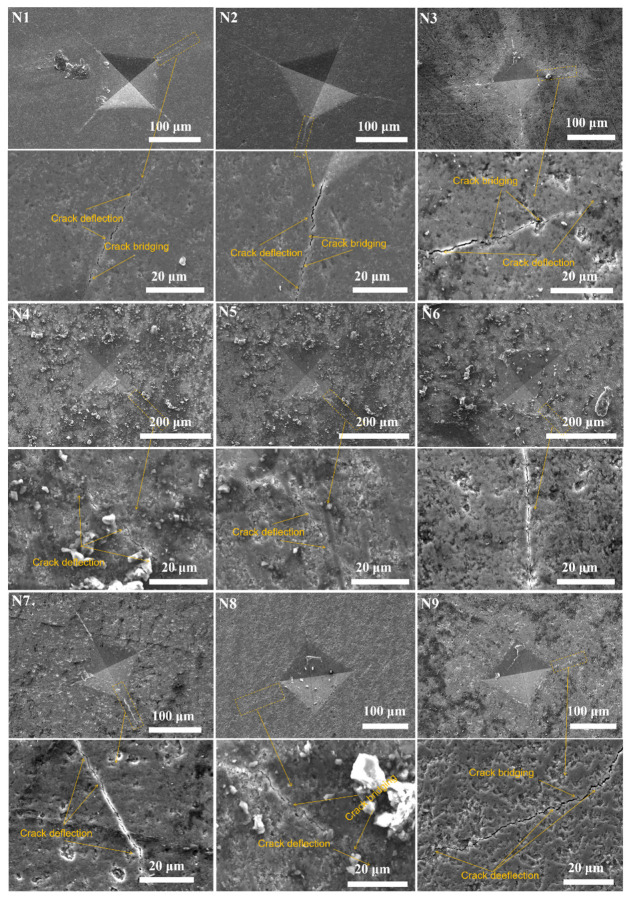
SEM micrographs of Vickers indents and crack propagation for samples N1–N9.

Figure 8 presents the bending strength, Vickers hardness, and fracture toughness of samples N1–N9, while Figure 9 explores the relationship between their microstructure and fracture toughness. The results indicate that the Si_3_N_4_ ceramics of samples N1–N3 outperform samples N4–N6 and N7–N9 in all mechanical properties. The elongated crystal structure of samples N1–N3 provides a hindering effect over a wide range, enhancing the overall strength of the material, with about 15% of the Ostwald ripening particles having larger grain diameters, significantly improving fracture toughness. Additionally, the elongated rod-like structures can span across cracks, forming “crack bridging”, which effectively transmits stress and suppresses crack propagation, further enhancing fracture toughness. However, as the amount of sintering additives increases, the number of elongated β-Si_3_N_4_ grains decreases, and the increase in silicon nitride grain size leads to a reduction in fracture toughness [39]. Sample N1 features a prominent cross-linked elongated β-Si_3_N_4_ rod-like crystal structure. When the long axis of these elongated grains aligns with the direction of applied stress, the material exhibits increased strength, as this orientation allows the long axis to effectively resist tensile and compressive stresses. Consequently, the strength of N1 surpasses that of N2, and N2 is stronger than N3, indicating that the elongated grain structure positively impacts the material’s overall mechanical properties. Moreover, as the alignment of the grain orientation with the stress direction improves, the overall mechanical performance of the material is further enhanced. This is because in ceramic materials, grains typically exhibit a multi-directional arrangement, and grains in different directions may show different deformation and fracture behaviors under external forces. If the grain orientation aligns with the applied stress direction, the slip or fracture process of the grains will occur more smoothly, reducing crack propagation and improving the material’s strength and fracture resistance. For samples N4-N6, the morphological images indicate that the number of rod-like β-Si_3_N_4_ crystals is limited, and the growth of β-Si_3_N_4_ grains has not fully reached the necessary conditions. This weakens the synergistic effects between the grains, resulting in reduced tensile and compressive strengths. Furthermore, as the content of sintering additives increases, grain agglomeration and size growth occur, further diminishing the mechanical properties of the material. Comprehensive analysis shows that the mechanical properties of sample N4 are superior to those of N5, and N5 outperforms N6 [40]. For samples N7 and N8, sample N7 demonstrates better mechanical properties, while an increase in the content of sintering additives leads to a decline in mechanical performance. SEM of samples N8 and N9 reveal a higher presence of liquid phases, which typically result in the formation of glassy or low-melting-point phases. These phases exhibit lower mechanical strength, and when distributed along the grain boundaries, they weaken the bonding forces between the grains. The weakening of grain boundaries not only reduces the material’s strength but also facilitates the initiation and propagation of cracks in fragile areas.

Based on the previous discussion and the results presented in Table 2, the comparison of samples N1–N9 indicates that the incorporation of Al_2_O_3_-Y_2_O_3_ sintering additives not only effectively improves the material density and bending strength but also optimizes the microstructure of Si_3_N_4_. Among all the samples, sample N1, which contains 5 wt% Al_2_O_3_-Y_2_O_3_ additives, exhibits the best bending strength, reaching 540.63 ± 10.05 MPa. Additionally, it has a hardness of 12.88 ± 0.52 GPa and fracture toughness of 4.92 ± 0.07 MPa·m^1/2^.

### 3.3. Crack Propagation Behaviors of Si_3_N_4_ Ceramic

**Figure 10:**
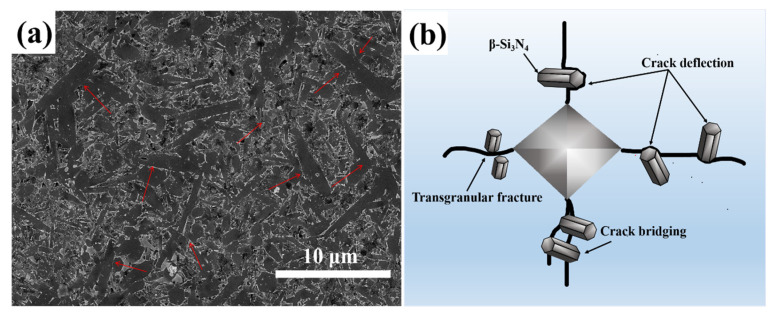
(**a**) SEM image of the N1 sample after 300 s of particle etching post gas pressure sintering; (**b**) Schematic diagram illustrating the fracture and toughening mechanisms present in the N1 sample.

Figure 9 shows the SEM images of Vickers indent crack propagation in ceramic samples N1–N9, obtained through indentation testing, detailing the toughening mechanisms of the samples. The cracks are magnified and examined to identify their propagation modes, with yellow arrows marking the locations of crack bridging and deflection. In most Si_3_N_4_ ceramic composites, a synergistic toughening effect is observed between the elongated β-Si_3_N_4_ grains and the sintering additive particles. This is manifested through various toughening mechanisms, including transgranular fracture, grain pullout, crack bridging, and crack deflection [41]. The images of N1, N2, and N3 clearly illustrate crack bridging and deflection, with samples displaying elongated rod-like grains. These elongated grains enhance fracture toughness through mechanisms such as crack bridging and deflection, indicating that the fracture toughness of samples N1-N3 is superior to that of the others. The grain pullout mechanism, however, is more complex and is significantly limited by the diameter of the needle-like grains. During the sintering process, needle-like Si_3_N_4_ grains grow, and densification causes collisions between adjacent grains. As the grains undergo phase transformation and growth, they intertwine, forming mutual mechanical locks, as shown by the arrows in Figure 10a. This interlocking reduces the likelihood of grain pullout, which explains the absence of significant pullout in Figure 9, where the primary toughening mechanisms are crack bridging and deflection [42]. Additionally, the thermal expansion mismatch between Al_2_O_3_/Y_2_O_3_ particles and the Si_3_N_4_ matrix induces interfacial fracture, resulting in residual stress around the reinforcing phase, which leads to greater twisting angles and significantly enhances the material’s fracture toughness. When the synergistic effect between the sintering additives and Si_3_N_4_ is low, such as with AlN sintering additives (N4, N5, N6), cracks primarily exhibit deflection. When these cracks reach β-Si_3_N_4_ grains, they require additional energy to continue propagating, resulting in crack arrest due to reduced driving force. Conversely, when Y_2_O_3_ and AlN are used together as sintering additives (N6, N7, N8), the presence of Y_2_O_3_ can enhance the fracture toughness of Si_3_N_4_, indicating that Y_2_O_3_ has toughening effects. However, the synergistic effect of Y_2_O_3_ and AlN is not as strong as that of Al_2_O_3_ and Y_2_O_3_, suggesting that adding Al_2_O_3_ can optimize the microstructure of Si_3_N_4_, thereby increasing the material’s density and mechanical properties. In summary, rod-like β-Si_3_N_4_ grains effectively impede crack propagation, extending the crack path and improving the toughness of Si_3_N_4_ ceramics. Among the additives, N1 exhibits the best synergistic effect, significantly enhancing the material’s resistance to crack propagation and fracture toughness. Moreover, crack fracture is the primary and effective toughening mechanism, rather than deflection and bending at the crack tip [43]. Figure 10b illustrates a detailed schematic of the toughening mechanism for the N1 sample.

## 4. Conclusions

1. The DLP 3D printing technology successfully prepared nine different formulations of Si_3_N_4_ ceramic green bodies with a solid content of 45 vol%. Using gas-pressure sintering, the samples were sintered for 2 h at 1 MPa and 1800 °C in an N_2_ atmosphere, resulting in the successful fabrication of dense Si_3_N_4_ ceramics with nine distinct formulations.
2. Morphological and grain distribution analyses revealed that samples with elongated β-Si_3_N_4_ grains exhibited fewer pores and a denser microstructure.
3. The addition of Al_2_O_3_-Y_2_O_3_ sintering aids enhanced the sintering capability of the Si_3_N_4_ samples and improved their mechanical properties. The sample containing 5 wt% Al_2_O_3_-Y_2_O_3_ exhibited the best sintering performance, achieving a Vickers hardness of 12.88 ± 0.52 GPa, flexural strength of 540.63 ± 10.05 MPa, and fracture toughness of 4.92 ± 0.07 MPa·m^¹/^_2_.
4. By integrating actual images with schematic illustrations of crack paths in the N1 sample, various toughening mechanisms in the Si_3_N_4_ ceramic composites were explored. Crack deflection and crack bridging within the Si_3_N_4_ matrix caused the crack tip to twist, which enhanced the fracture toughness. The increased obstacles presented by the elongated β-Si_3_N_4_ grains and Al_2_O_3_/Y_2_O_3_ particles contributed to the N1 sample, demonstrating a richer array of fracture and toughening pathways.

Main Advantage: This study successfully fabricated nine different formulations of Si_3_N_4_ ceramics using DLP 3D printing technology and achieved densification through gas-pressure sintering in a nitrogen atmosphere. The addition of 5 wt% Al_2_O_3_-Y_2_O_3_ sintering aids effectively improved the sintering performance, enhanced the microstructure, and improved the mechanical properties of the materials. The toughening mechanisms in Si_3_N_4_ ceramic composites were investigated, revealing the contributions of crack deflection and bridging to the improvement of fracture toughness, which further enriched the toughening pathways of the material. This provides an effective solution for the application of high-performance ceramic materials.

## 5. Outlook

Future research could build upon this study by further optimizing the sintering temperature and systematically investigating the effects of various multicomponent (e.g., ternary, quaternary, or quinary) sintering additives on the performance of Si_3_N_4_ ceramics, particularly concerning their combined improvements in material density, mechanical properties, thermal shock resistance, and oxidation resistance. Additionally, for the application of DLP 3D printing technology, future studies should focus on enhancing printing precision and efficiency while exploring methods for fabricating more complex geometries and functional ceramic components. This will help expand the potential applications of Si_3_N_4_ ceramics in customized and high-precision manufacturing fields.

Considering the wide-ranging applications of Si_3_N_4_ ceramics in critical industries such as aerospace, semiconductors, healthcare, automotive, and energy, future research should also address the specific requirements of these fields by conducting performance tests and optimizations under extreme environmental conditions. For instance, long-term stability tests in high-temperature, corrosive atmospheres, and radiation environments can provide more targeted material design and improvement strategies for Si_3_N_4_ ceramics in these industrial applications.

Finally, while this study explored the role of crack deflection and bridging mechanisms in enhancing fracture toughness, future research could further leverage advanced microstructural analysis techniques, such as Transmission Electron Microscopy (TEM) and Atomic Force Microscopy (AFM), to uncover additional toughening mechanisms. This will facilitate a deeper understanding of the material’s fracture behavior at the microscopic level and enable more refined material design, thereby further enhancing the overall performance of Si_3_N_4_ ceramics and advancing their widespread use in high-end applications.

## Figures and Tables

**Table 1:** Compositions of samples.

Sample	Si_3_N_4_ (wt%)	Y_2_O_3_ (wt%)	Al_2_O_3_ (wt%)	AlN (wt%)
N1	95	2.5	2.5	_
N2	90	5	5	_
N3	85	7.5	7.5	_
N4	95	_	2.5	2.5
N5	90	_	5	5
N6	85	_	7.5	7.5
N7	95	2.5	_	2.5
N8	90	5	_	5
N9	85	7.5	_	7.5

**Table 2:** Phase content, mechanical properties, and relative density of Si_3_N_4_ ceramics composites.

Sample	N1	N2	N3	N4	N5	N6	N7	N8	N9
Flexural strength/MPa	540.63 ± 10.05	513.12 ± 24.23	507.64 ± 15.35	142.95 ± 20.21	129.34 ± 15.23	115.67 ± 10.15	377.28 ± 25.37	359.62 ± 18.8	321.84 ± 10.9
Vickers hardness/GPa	12.88 ± 0.52	11.73 ± 0.30	11.59 ± 0.15	5.09 ± 0.20	3.12 ± 0.23	2.60 ± 0.15	14.19 ± 0.36	11.34 ± 0.30	8.89 ± 0.10
Fracture toughness/MPa·m^1/2^	4.92 ± 0.07	4.20 ± 0.02	4.09 ± 0.07	4.67 ± 0.01	4.32 ± 0.02	3.85 ± 0.04	4.70 ± 0.03	4.59 ± 0.01	4.55 ± 0.02
Relative density/%	84.2 ± 10.0	83.91 ± 20.02	81.5 ± 10.08	71.18 ± 30.07	70.63 ± 20.58	63.4 ± 20.03	80.3 ± 11.70	77.4 ± 12.25	70.0 ± 21.4

## Data Availability

The data supporting this study’s findings are available from the corresponding author upon reasonable request.

## Supplementary Materials

The following supporting information can be downloaded at: https://www.mdpi.com/article/10.3390/ma17235830/s1, Figure S1: Schematic diagram of the (**a**) degreasing and (**b**) sintering process of nine types of Si3N4 samples.
