# Supplementary material for: Impact of Sintering Aid Type and Content on the Mechanical Properties of Digital Light Processing 3D-Printed Si3N4 Ceramics
Source: Materials (Basel). 2024 Nov 27;17(23):5830. doi: 10.3390/ma17235830 (PMC11642628; doi:10.3390/ma17235830)
Supplement: Supplementary file 1 [file materials-17-05830-s001.zip › materials-3319103-supplementary.pdf]

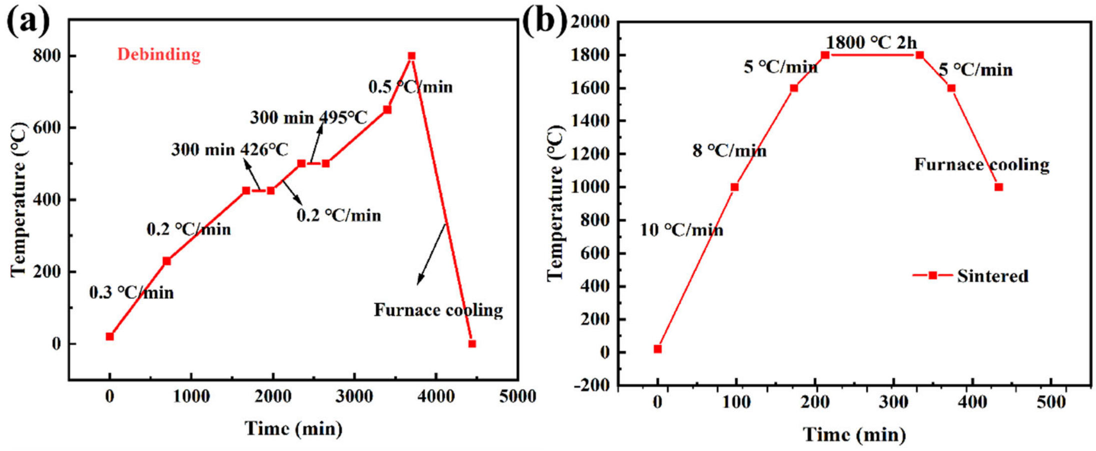

Figure S1. Schematic diagram of the (a) degreasing and (b) sintering process of nine types of Si_3N_4 samples.

The Si_3N_4 green body printed using DLP technology is thoroughly cleaned with alcohol, and then placed in a muffle furnace (Luoyang Guoju Precision Electric Furnace Co., Ltd., China) to remove the photosensitive resin before sintering. The debinding curve and sintering process are shown in Fig. S (a) and (b), respectively. First, the printed green body is heated from room temperature to 224°C at a constant rate of 0.3°C/min, then raised to 426°C at a rate of 0.2°C/min and held for 300 mins, followed by an increase to 495°C, also held for 300 mins. Next, it is heated to 550°C at a rate of 0.2°C/min, and finally to 800°C at a rate of 5°C/min, with cooling occurring alongside the furnace.

After debonding, to explore the effects of different sintering temperatures on the microstructure and properties of the ceramics, the debound Si_3N_4 green body is sintered in the high-temperature pressure sintering furnace (Henan High-Temperature Technology Group Co., Ltd., China) at a specific rate. The heating rate is 10°C/min to 1000°C, then 8°C/min to 1600°C, and finally 5°C/min to 1800°C, maintained for 2 h, all under a constant nitrogen atmosphere of 1 MPa, resulting in Si_3N_4 ceramics.
